# A randomized comparative effectiveness trial to evaluate two programs for promotion of physical activity after spinal cord injury in manual wheelchair users

**DOI:** 10.3389/fspor.2025.1504840

**Published:** 2025-02-12

**Authors:** Jenna M. Martinez, Lisa L. Haubert, Valerie J. Eberly, Walter B. Weiss, Jeffery W. Rankin

**Affiliations:** ^1^ Pathokinesiology Laboratory, Rancho Los Amigos National Rehabilitation Center, Downey, CA, United States; ^2^ School of Medicine, University of California, Irvine, CA, United States

**Keywords:** spinal cord injury, physical activity, exercise, cardiometabolic health, paraplegia, randomized control trial

## Abstract

**Objective:**

The goal of this study was to determine the effectiveness of a novel whole of day activity accumulation (WODAA) physical exercise program. WODAA physical activity and physiological outcomes were compared to outcomes from individuals using a traditional planned arm crank exercise (PACE) program. Both programs included progressive exercise instruction and goal setting over a 4-month period, and utilization of a wrist-worn activity monitor (Fitbit Blaze/Versa, Fitbit Inc., San Francisco, CA).

**Design:**

Longitudinal, randomized, comparative effectiveness trial with collaborative goal setting.

**Setting:**

Research laboratory at a rehabilitation hospital and in participants' homes and communities.

**Participants:**

Forty-nine manual wheelchair users with paraplegia.

**Outcome measures:**

Physical activity measurements and cardiometabolic data were collected before, during, and after the program. The primary measures were amount of daily arm activity (Steps) and time spent in different activity and heart rate zones.

**Results:**

Relative to baseline measures, participants in the WODAA group had significantly more daily arm movement/propulsion activity (Steps) and time spent in the Fairly and Very Active Zones and the Cardio Heart Rate Zone compared to those in the PACE group over the final month of the intervention (*p* < 0.05). Minutes spent in other Activity and Heart Rate Zones were similar between groups. At final evaluation, diastolic blood pressure after a 6-Minute Push Test was significantly lower in the WODAA group, while no differences were found in distance traveled, systolic, or pre-test diastolic blood pressures. Metabolic bloodwork and shoulder pain scores did not change and were similar between groups.

**Conclusion:**

Depending on the measure used, these findings suggest that a WODAA approach to PA is comparable or more effective than a traditional PACE program in promoting physical activity in low-active manual wheelchair users with paraplegia.

## 1 Introduction

Physical activity (PA), including muscle strength training and aerobic exercise, in adults with or without a disability, is critical for preventing major chronic health conditions such as Type 2 diabetes and cardiovascular disease (1). Despite the well-documented physical and psychological benefits of PA, activity levels remain low in persons with spinal cord injury (SCI) (2–5). Individuals with mobility limitations (including SCI) engage in significantly less PA than the general population and are less likely to attain recommended levels of moderate and vigorous PA (6–8). Moreover, those who use manual wheelchairs (MWCs) spend significantly more time in sedentary activities, an independent risk factor for poor health, compared to non-disabled individuals (9). In addition, inactivity in persons with SCI contributes to abnormalities in carbohydrate and lipid metabolism, higher prevalence of diabetes mellitus, and an earlier occurrence of coronary heart disease and stroke relative to the general population (10, 11). Indeed, Cragg and colleagues concluded that these consequences of inactivity point to an “exigent need for targeted interventions and prevention strategies addressing modifiable risk factors for cardiovascular disease in individuals with SCI” (11).

A dilemma for those living with SCI who use an MWC is determining how to increase PA for physical and psychological health benefits without further contributing to the largely untreated problem of shoulder pain and dysfunction that negatively impacts mobility, participation, and quality of life (12). In addition, traditional forms of aerobic exercise for these individuals such as planned arm-crank ergometry (PACE) are often associated with barriers to sustainability including lack of time, resources (transportation to gyms and cost of membership or exercise equipment), and limited availability of accessible exercise facilities and equipment (13). Thus, identifying a program for those with SCI to circumnavigate the barriers associated with traditional PACE programs is critical to increasing and sustaining PA in order to obtain the associated health benefits.

One promising alternative to traditional aerobic exercise is the use of a whole of day activity accumulation (WODAA) approach. The WODAA approach is designed to decrease sedentary time and increase overall PA by measuring and accumulating activity throughout the day (9, 14, 15). WODAA has been demonstrated as effective in improving PA in the non-disabled population (14), but its efficacy has not been clearly established in other groups (9, 15). By expanding the bounds of where, when, and how movement or PA can be performed throughout the day, this intervention could more effectively address the health needs of persons with disabilities, including individuals from traditionally underserved populations with limited financial resources, by alleviating many of the barriers to exercise for persons with SCI who use an MWC. Additionally, this approach could help alleviate concerns about increasing the incidence of shoulder pain or overuse syndromes (16–18) by dispersing rest and PA bouts throughout the day.

Commercially available activity monitors have great potential as an intervention support and data collection tool. Previous work has demonstrated that using these devices to implement feedback increased PA in an underserved nondisabled population (19), suggesting that they also could be used to improve the effectiveness of our novel WODAA PA intervention. If it can be demonstrated that these devices are able to support PA programs for MWC users with SCI, then the widespread prevalence of these devices could facilitate long term adoption and improved ability to track progress towards and achievement of daily PA goals that may better address the health needs of persons with disabilities when compared to traditional PA programs. In addition, if found to be sufficiently accurate, these devices may also be used as a research/data collection tool to support the documentation of home- and community-based PA. To this end, we pilot tested two of the activity monitors readily available at the time of data collection (Fitbit Charge, Fitbit Inc., San Francisco, CA; Garmin Vivofit, Garmin International, Inc. Olathe, KS) to evaluate their ability to accurately collect wheelchair-based activity data. Two manual wheelchair users with paraplegia wore the devices on their wrists while performing several common arm activities, with recorded Fitbit values compared to manual counts/measures. We determined that the Fitbit Charge was sufficiently accurate for recording arm movements across all three exemplary tasks: (1) a 15-min bout of arm cycling (4.4% error), (2) maneuvering indoors with slow, sporadic pushes (11.5% error), and (3) 10 repetitions each of forward arm elevation (shoulder flexion) to 90° and 180° and reaching to the side (shoulder abduction) to 90° (6.7% error). In general, the Fitbit underestimated arm activity except during arm cycling, where the device exhibited a small overestimation of cycles performed (1,341 vs. 1,285 actual or 4.4% error). Based on this pilot work, we concluded that a Fitbit (in this case the Blaze and its subsequent replacement, the Versa) was sufficiently accurate to be investigated further in our activity-based intervention and as a data collection tool.

The goal of this study was to compare the ability of a novel 16-week WODAA PA program to increase PA and improve cardiometabolic health relative to a traditional PACE program in persons living with SCI who use an MWC for mobility. We hypothesized that, relative to PACE, the WODAA program would result in (1) a greater increase in PA and (2) more substantial improvements in cardiometabolic health measures (e.g., insulin resistance). We also hypothesized that both intervention groups would not experience significant increases in shoulder pain.

## 2 Materials and methods

### 2.1 Participants

A convenience sample was recruited based on the following inclusion criteria: having paraplegia resulting from an SCI [American Spinal Injury Association Impairment Scale (AIS) A-C (20)] for at least 1 year; ≥18 years of age; uses an MWC for community mobility; interested in increasing their PA. Participants were also asked if they were currently exercising or playing sports. Individuals that responded in the affirmative were excluded if their participation in regular aerobic exercise or sports was ≥3 times weekly for 30 min or more per session. Additional exclusion criteria included: history of upper extremity surgery in the past year; physician-recommended limits on PA; cardiac abnormalities found on electrocardiogram (ECG) screen precluding maximal exercise testing; shoulder pain limiting MWC propulsion; full-thickness/large rotator cuff tear; pregnancy or planning to become pregnant in near future.

**Figure 1:**
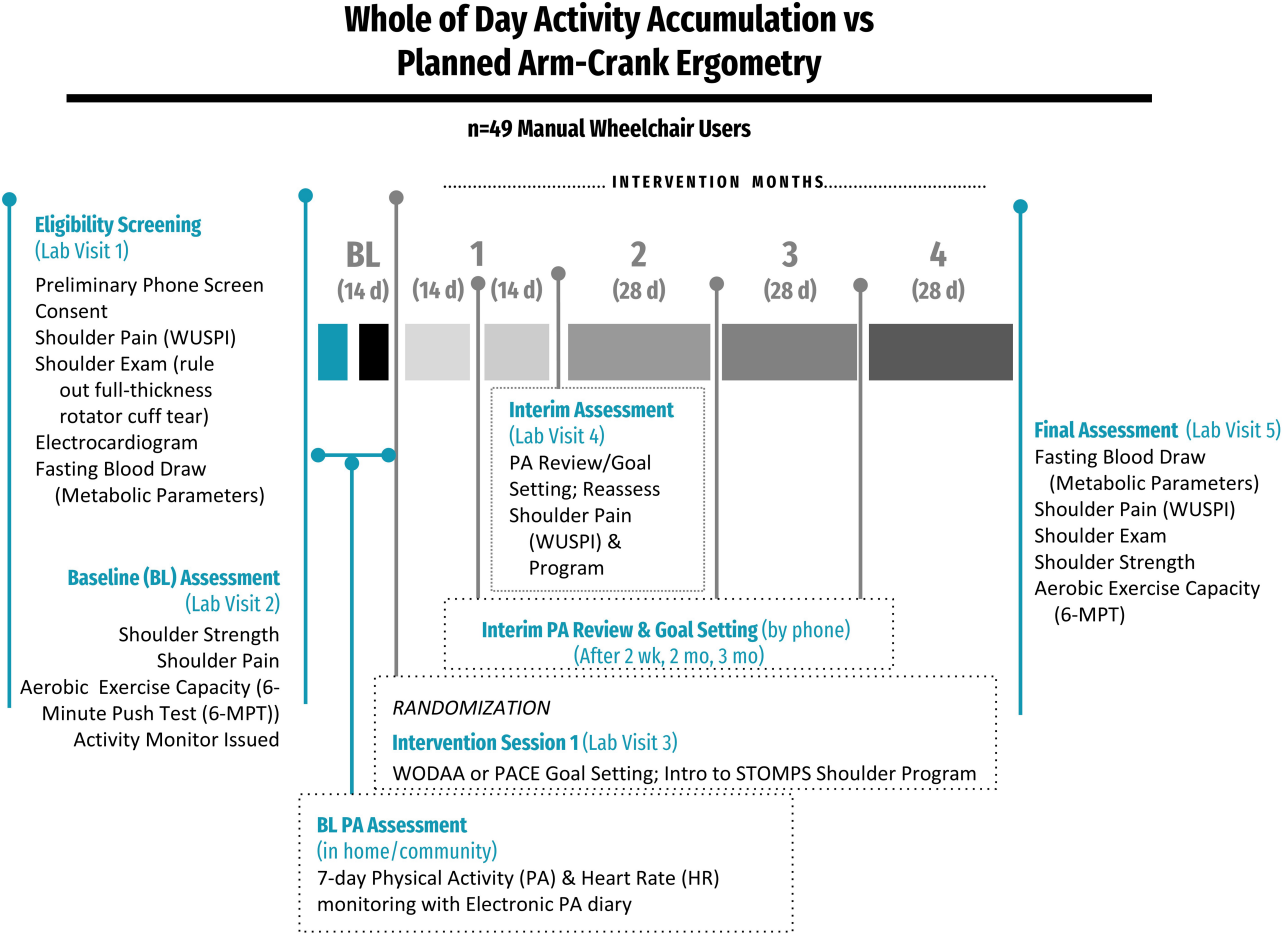
Overview of the study design. After Baseline (BL) assessments, participants were randomized into either the Whole of Day Activity Accumulation (WODAA) or Planned Arm Crank Ergometry (PACE) intervention groups. After randomization, interim assessments were performed every two weeks in the first month and monthly (every 28 days) thereafter.

Prior to participation, volunteers reviewed, signed, and received a copy of the Bill of Rights of Human Subjects and informed consent form approved by the Rancho Research Institute Institutional Review Board. Following screening for eligibility, the study was conducted over 5 in-person sessions at the Pathokinesiology Laboratory at Rancho Los Amigos National Rehabilitation Center (RLANRC): one Screening, three Assessment (Initial, Interim, and Final), and one Training/Intervention Session. In addition, participant PA and Heart Rate (HR) data were collected through remote monitoring using a wrist-worn activity-tracking device (Fitbit Blaze/Versa, Fitbit Inc., San Francisco, CA) during home and community activity over a 7–14 day Baseline Assessment and 4-month-long intervention. Interim PA review and goal setting sessions also occurred via phone after 2 weeks, 2 months, and 3 months of the intervention (Figure 1).

### 2.2 Instrumentation

#### 2.2.1 Metabolic laboratory tests

Blood samples were collected and analyzed to obtain a fasting lipid profile with its fractions as well as fasting glucose, insulin, and C-reactive protein levels. Homeostatic model assessment (HOMA-1 and HOMA-2) scores estimated beta-cell function (%B) and insulin sensitivity (%S) (21, 22). Insulin resistance (HOMA-IR) was assessed via the model index, where:

(1) $$\mathrm{Model}\;\mathrm{Index}\;=\;\mathrm{insulin}\left[\frac{\mu \mathrm{IU}}{\mathrm{mL}}\right]\times \;\frac{\mathrm{fasting}\;\mathrm{glucose}\left[\frac{\mathrm{mmol}}{L}\right]}{22.5}$$

#### 2.2.2 Wheelchair User's Shoulder Pain Index (WUSPI)

The Wheelchair Users Shoulder Pain Index (WUSPI) is a survey instrument consisting of 15 items that measures the amount shoulder pain the respondent experienced over the last 7 days while performing functional activities (23). The instrument has been documented to be highly reliable with repeated administration (*r* = 0.9), as well as having good internal consistency, and concurrent validity when utilized to indicate the effects of intervention on shoulder pain (23).

#### 2.2.3 Six-Minute Push Test (6-MPT)

The 6-MPT was developed as a clinically practical test of aerobic capacity for persons with SCI. It has a high test-retest reliability [ICC of 0.97 (95% confidence interval: 0.93–0.99)] and, in persons with paraplegia, demonstrates a strong correlation with peak oxygen consumption values elicited during a maximum arm-crank ergometry test [ICC of 0.86 (0.70–0.93)] (24, 25). As a result, the 6-MPT is able to distinguish between fitness levels in those with paraplegia (24) and was utilized in this study to assess aerobic exercise capacity and fitness. Participants were instructed to push their usual MWC as many laps as possible around a track of known distance in 6 minutes and were allowed to set their own pace and permitted to take rest breaks, if needed. Participants were encouraged in their efforts at the end each minute. Blood pressure and heart rate were recorded just prior to and at the end of the 6-min period and the total distance covered during the 6-MPT was recorded. Participants also wore a heart rate monitor (Polar H10, Polar Electro Inc., Bethpage, NY) during the assessment to obtain average and peak heart rate (24).

#### 2.2.4 Daily physical activity (PA) & heart rate (HR) data

Home and community PA data were collected using a wireless activity monitor worn on the dominant wrist (Fitbit Blaze/Versa, Fitbit Inc., San Francisco, CA). The device documented MWC propulsion/arm movement as well as continuous heart rate. Arm activity data were measured using the devices “Steps” recording feature, with our pilot data demonstrating that each wheelchair push registered approximately 2 Steps in the wrist-worn activity monitor and that a 30-min bout of arm crank ergometry exercise contributes an additional 800–1,500 Steps per day. To validate this approach, we compared the activity monitor reported propulsion cycles (Steps completed) to a direct push count obtained during the baseline 6-MPT in a subset of 11 participants. We found the validity coefficient to be 0.90 (*p* = 0.000), with the mean absolute percentage error (MAPE) of 10.5% (SD 11.4%), just slightly above the established standard acceptable MAPE of 10% (26). We additionally analyzed the accuracy of the wrist-worn activity monitor HR by comparing its HR output to a Polar HR monitor (H10, Polar Electro Inc., Bethpage, NY) during each minute of the first PACE intervention session. We found excellent validity with significant ICCs ranging from 0.81–0.98 (*p* = 0.000) and MAPEs ranging from 1.9% to 4.9%, well below the acceptable threshold.

### 2.3 Procedures

#### 2.3.1 Eligibility screening

Interested individuals were initially screened via telephone or in-person to determine preliminary eligibility (Figure 1). Potential participants were consented and the presence of shoulder pain and/or likely subacromial structure pathology was then documented using the WUSPI and performance of a clinical shoulder exam by a licensed physical therapist. The clinical shoulder exam included documentation of bilateral maximum active and passive shoulder abduction and external rotation (at 90° of shoulder abduction and elbow flexion and neutral forearm pronation/supination) range of motion and clinical test results for subacromial impingement syndrome/rotator cuff tendinopathy [Supraspinatus test (Empty Can); Hawkins-Kennedy Impingement test, External Rotator Strength test, and Codman's Drop Arm Test]. If individuals were free of shoulder pain (WUSPI score ≤12) and the clinical shoulder exam indicated a minimal likelihood of subacromial impingement syndrome/rotator cuff tear (27, 28), a fasting blood draw was performed for cardiometabolic analysis and an ECG was collected and interpreted by a cardiologist to ensure that the individual did not have a condition that would preclude them from increasing PA.

#### 2.3.2 Baseline assessments

Once labs and ECG were reviewed and the participant was cleared by a cardiologist, the WUSPI was again completed and participants were asked to complete a 6-MPT. They were provided with a wrist-worn wireless activity monitor with the screen covered by an opaque black film to prevent participants from receiving device PA feedback. Participants were instructed to wear the device on their dominant wrist. The Fitbit mobile app was installed on a phone (participant's personal or a borrowed lab phone) for remote data acquisition and monitoring. Participants were instructed to not tamper with the occlusive screen cover and continue customary PA for the duration of the Baseline period. They were instructed to wear the device during waking hours (at least 8 h per day) and to charge the device while sleeping or bathing. The Baseline data collection period lasted 10–14 days, where data were collected in the participant's home and community environment. The period was selected to allow for a full 7 days of PA data acquisition with the first 7 complete days of typical activity used in the analysis.

#### 2.3.3 Initial intervention visit

Following the Baseline home/community PA collection period, individuals returned to the lab and were randomized into either the (1) Whole of Day Activity Accumulation (WODAA) or (2) Planned Arm Crank Ergometry (PACE) intervention groups. Randomization was performed using a pre-populated chart consisting of random numbers where each entry had either a 1 or 2. Following screening, enrollment, and baseline PA assessment, participants were sequentially assigned to the next entry on the random numbers chart, with the corresponding number indicating the participant's designated intervention group (1 = WODAA; 2 = PACE). However, due to the nature of the study intervention, randomization was blocked by groups of five such that if one intervention group block became full with five participants, subsequent enrollees were forced into the opposite intervention group until it was then capped at five enrollees (29). In this case, participants were assigned the next available entry in the random numbers chart that corresponded to their forced group. Participants in the WODAA group reviewed their baseline 7-day PA and HR data with a physical therapist to understand their current PA habits. For participants in the WODAA group the occlusive activity monitor screen cover was removed, and they were educated in how to use the wrist-worn activity monitor and phone app to view and track their PA and HR. Individuals in the PACE group were provided an arm crank ergometer for home use throughout the intervention. They were asked to log each exercise session date, duration, distance, and maximum resistance upon completion into a data-logging phone app. Their opaque screen cover remained in place on their wrist-worn activity monitor, with only HR feedback displayed during arm-crank ergometry sessions on the phone app to display exertion feedback to support attainment of exertion-based arm-crank goals.

Both groups received individualized goal-setting with a physical therapist utilizing the Brief Action Planning technique for collaborative PA goal setting and plan achievement design (30, 31). Goals were tailored to either the PACE or WODAA intervention and the individual's current PA level and upper extremity health. Participants were encouraged to take the lead on goal setting, though standardized goals and associated progression metrics were suggested as needed to ensure each participant set challenging but realistic goals. Initial goals were set during the initial intervention visit and progressively updated following Week 2 and Months 1, 2, and 3 of the intervention. As part of goal setting and attainment review, participants were also asked to subjectively report their level of exertion during PA using Borg Rating of Perceive Exertion (RPE) Scale (32). The RPE is a 6-to-20-point scale that is widely used to guide exercise intensity, with higher numbers associated with higher intensity activities. Goals for the WODAA group focused on progressively increasing daily PA (Steps/arm activity) to decrease sedentary time. Depending on the participant's starting capacity, goals followed the general progression framework: (1) average at least 10,000 Steps/day, (2) increase the number of hours each day with at least 250 Steps (once 10,000 daily Steps were consistently achieved), (3) increase the amount of time spent in higher heart rate zones (i.e., the time reported by the activity monitor in the Cardio HR Zone and/or the time spent exercising at a participant reported RPE ≥12). Goals for the PACE group were also progressive in nature. Initially, participants were asked to perform three 15-min cycling sessions each week at a target heart rate of at least 70% of the calculated maximum rate (typically corresponding to a participant reported RPE of 12–16). All PACE sessions included a 2-min warm-up and 1-min cool-down. PACE participants were then encouraged to progressively increase session duration from 15 to 30 min between Weeks 2 and 4 and to 33 min by Week 5. Last, participants were instructed to maintain 33–35 min sessions, but exercise at a higher intensity (target heart rate of 85% of maximum). They were encouraged, but not required, to disperse their three PACE sessions across the 7 days each week and advised to increase resistance and speed during Weeks 5–12 to meet these established goals. If participants achieved the Weeks 5–12 goal, they were encouraged to maintain that level in Weeks 12–16.

Both groups received equipment and instruction on performance of the STOMPS shoulder preservation program. Participants were encouraged to perform the STOMPS strengthening exercises three times a week, with a day of rest between resistance sessions, throughout the study. The program consists of home-based shoulder flexibility and strengthening exercises and recommendations for movement techniques that reduce shoulder demands associated with PA and daily activities after SCI (29, 33, 34).

#### 2.3.4 Interim and final assessments

During interim assessments, participants had either an in-person or telephone appointment with the physical therapist to evaluate progress towards achieving their PA goals and, if appropriate, to progress their goals. Between assessments participants continued with either their WODAA or PACE intervention in their home and community environments. During the 2-week phone assessment, goals were set for the end of Month 1. Month 1 review and goal setting was performed in-person, where participants also received additional assistance related to study equipment and device use and progression of their shoulder strengthening program as appropriate. Shoulder pain status was also formally assessed. Two additional phone assessments, which included the review of previous goal achievement and setting new monthly goals occurred at the end of Months 2 and 3. Upon completion of the intervention (end of Month 4), a fasting blood draw was again conducted to obtain cardiometabolic variables. Participants also repeated the 6-MPT and completed the WUSPI questionnaire as well as the clinical shoulder exam, if pain was indicated on the WUSPI. Participants were permitted to keep their activity monitors upon program completion. Individuals in the PACE group were instructed in use of the wearable activity monitor and mobile app, if desired.

#### 2.3.5 Additional contact

Outside of scheduled visits, contact between the study team and participants occurred as needed. Participants occasionally reached out for technical support/assistance with equipment and device setup. In addition, prolonged breaks in activity monitor data (≥3–5 days), including identified data syncing issues, prompted the research team to contact participants.

### 2.4 Data management

Metabolic lab values and 6-MPT data from the Baseline and Final (Month 4) program assessments were logged for analysis. Shoulder pain (WUSPI) was evaluated at the Screening, Baseline, intervention Month 1 and Final Assessments as well as at any other time a reported change in shoulder symptoms and/or injury occurred.

Individual participant daily PA data was exported from the Fitbit website and conglomerated into averages for the Baseline period (7 days) and monthly intervention periods (Months 1–4). Heart rate (HR) data were additionally recorded from the Fitbit portal. Daily PA and HR metrics were routinely monitored and screened every 3–5 days for full days of use (≥8 waking hours) to ensure adequate device function and that data represented the majority of a participants' activity. Only days with ≥8 waking hours and only months with over half of the days (≥14 days) with valid data were utilized for data analysis. Activity data were binned using Activity Zones, which were defined by estimating the Metabolic Equivalent (MET) of an activity based on the commercial activity monitor's measurement of resting and current HR. Sedentary, Light, Fairly Active, and Very Active Zones were defined using the ≤1, 2–3, 4–6, and >6 METs thresholds, respectively, that are established for able-bodied individuals (35). Activity was also binned based on the recorded heart rate data, which included time spent in the Fat Burning, Cardio, and Peak Heart Rate Zones. These zones were defined as 50%–69%, 70%–84% and 85%–100% of maximum calculated heart rate. To obtain an estimation of time spent in various heart rate and activity zone levels, the maximum heart rate (HR_max_) for each participant was entered into the activity monitor prior to participant issuance according to the equation (36):

(2) $$HR_{\max}=(208\;\text{-}\;(0.70\;\times \;\mathrm{age}))$$

Regardless of group, participants were placed on a PA intervention hold if they experienced a health condition that affected their ability to exercise for more than 3 consecutive days or if they had a malfunctioning activity monitor. In these cases, the equivalent amount of time was added to the duration of their intervention period. Holds lasted between 3 and 17 days, except for one participant who developed an infection and was unable to exercise for 11 weeks. Placing individuals on an intervention hold may have induced detraining effects, especially for those with longer holds. However, in this study, only the secondary (cardiometabolic) measures might be influenced by detraining, as our primary metrics [activity levels (Steps), HR and Activity Zones] are instantaneous measures of activity as opposed to observations of long-term physiological changes. While previous work has found detraining effects to occur (37–39), the amount of detraining time needed before an individual living with SCI experiences physiological changes can vary greatly (from 1 to more than 16 weeks) depending on the metric measured, with most time periods greater than the holds required by the participants in this study. For example, Gurney et al. (39) estimated that improvements in VO2 and HR measures were partially retained even after 8 weeks of detraining, while Gorgey et al. (38) did not observe any changes in basal metabolic rate, insulin sensitivity, and resting blood pressure after 16 weeks (although they observed decreases in muscle mass and cross-sectional area).

### 2.5 Statistical analysis

Complete data sets were analyzed using SPSS Statistics (Version 23) software (IBM, Chicago, IL, USA). After testing for normality (Shapiro–Wilk test), differences between and within groups of normally distributed data were assessed using a two-way analysis of variance with repeated measures and Tukey's *post hoc* tests. Analyses of nonparametric data within intervention groups were performed with Wilcoxon signed-rank tests. The Independent-Samples Mann–Whitney *U*-Test was utilized for nonparametric comparisons between intervention groups. The significance level for all tests was set to *p* < 0.05. Due to multiple group and timepoint comparisons (Group: WODAA vs. PACE, and Timepoint: Baseline vs. Month or Initial vs. Final Eval) a Bonferroni correction for multiple comparisons was applied for *post hoc* statistical tests.

Primary PA outcome measures were: (1) Daily average activity monitor data (Steps, Activity Zone minutes, HR Zone minutes) at Baseline and Month 4. The difference between the daily average of the 7-day Baseline and the daily average of the final month of the intervention were used to assess long-term change. Secondary PA measures included (1) fasting metabolic bloodwork (lipid profile, calculated HOMA-IR insulin resistance, glucose) and (2) 6-Minute Push Test distance traveled and blood pressure. Changes in Wheelchair User's Shoulder Pain Index (WUSPI) scores were also assessed.

## 3 Results

### 3.1 Participants

Fifty-four (24 WODAA, 30 PACE) of 65 qualifying participants completed the entire protocol (4-month intervention and Final Assessment). Of those completing the intervention, 5 participants (3 WODAA, 2 PACE) had insufficient PA data available for analysis, resulting in 49 (21 WODAA, 28 PACE) complete data sets for the current analysis. There were no significant differences between the two groups with respect to age and time since injury (Table 1), with the average age of all participants 40.8 years (range 21.7–60.9 years) and average injury duration 16.6 years (range 1.4–38.0 years). Other baseline demographic characteristics were statistically similar between the WODAA and PACE participants, as were initial cardiometabolic laboratory test values (Table 1). Baseline PA levels including average daily Sedentary, Light, Fairly, and Very Active Zone minutes as well as Fat Burn, Cardio, and Peak HR Zone minutes were also similar between the two groups (Table 2). No individual reported having shoulder pain on their initial WUSPI assessment (Table 1).

**Table 1:** Self-disclosed participant demographics.

Variable	Participant group					
	WODAA (*n* = 21)			PACE (*n* = 28)		
	Total	High para (T2-T6)	Low para (T7-L3)	Total	High para (T2-T6)	Low para (T7-L3)
Age, years (range)	40.9 (26.8–57.4)	34.4 (26.8–48.3)	44.8 (32.4–57.4)	40.7 (21.7–60.9)	46.6 (27.8–60.9)	38.3 (21.7–59.7)
Sex, % Female	5/21 (24%)	1/8 (13%)	4/13 (31%)	5/28 (18%)	2/8 (25%)	3/20 (15%)
Duration of injury, years (range)	14.1 (1.4–38.0)	8.7 (1.9–17.0)	17.4 (1.4–38.0)	18.4 (1.4–34.0)	24.1 (4.8–34.0)	16.1 (1.4–32.4)
Level of injury	*n* = 21	8 (38%)	13 (62%)	*n* = 28	8 (29%)	20 (71%)
AIS completion score						
A	15 (72%)	7 (88%)	8 (62%)	16 (57%)	4 (50%)	12 (60%)
B	3 (14%)	1 (12%)	2 (15%)	8 (29%)	4 (50%)	4 (20%)
C	3 (14%)	0 (0%)	3 (23%)	4 (14%)	0 (0%)	4 (20%)
Race/Ethnicity						
American Indian	0 (0%)	0 (0%)	0 (0%)	0 (0%)	0 (0%)	0 (0%)
Asian/Pacific Islander	1 (5%)	0 (0%)	1 (8%)	2 (7%)	1 (12%)	1 (5%)
Black	3 (14%)	1 (12%)	2 (15%)	6 (22%)	3 (38%)	3 (15%)
White	14 (67%)	6 (76%)	8 (62%)	13 (46%)	3 (38%)	10 (50%)
Unknown/Declined	0 (0%)	0 (0%)	0 (0%)	2 (7%)	1 (12%)	1 (5%)
More than one race	3 (14%)	1 (12%)	2 (15%)	5 (18%)	0 (0%)	5 (25%)
Ethnicity, % Hispanic	11/21 (52%)	5	6	19/28 (68%)	5	14
Avg. yearly income						
$0–25,000	16 (76%)	6	10	25 (89%)	7	18
$25,001–50,000	4 (19%)	2	2	3 (11%)	1	2
$50,001–75,000	0 (0%)	0	0	0 (0%)	0	0
≥$75,000	1 (5%)	0	1	0 (0%)	0	0
Self-described exerciser, yes	12 (57%)	4 (50%)	8 (62%)	14 (50%)	4 (50%)	10 (50%)
Self-reported cardiometabolic-related medical history	2 (10%)	0 (0%)	2 (15%)	5 (18%)	1 (13%)	4 (20%)
Baseline daily steps	5,992 ± 2,042	4,881 ± 2,093	6,675 ± 1,750	5,200 ± 2,298	5,496 ± 2,530	5,081 ± 2,257
Baseline sedentary time (min/day)	1,072 [814–1,194]	1,156 [1,000–1,232]	1,026 [785–1,084]	1,068 [770–1,144]	674 [638–963]	1,111 [938–1,139]
Baseline wheelchair user's shoulder pain index (WUSPI) score (pain ≥12)	0 [0, 0]	0 [0, 0]	0 [0, 0]	0 [0, 0]	0 [0, 0]	0 [0, 0]

Data are presented as overall values (Total) for each intervention group (WODAA, PACE) as well as broken down into High Paraplegia (T2-T6; High) and Low Paraplegia (T7-L3; Low) subgroups. Values are presented as counts (percent %), mean ± 1SD or median [25%–75% Interquartile Range].

**Table 2:** Group average daily minutes spent in the various activity levels and heart rate zones.

	Baseline (7 days)				Final intervention month (month 4)				Change			
	WODAA (*n* = 21)	PACE (*n* = 28)	t(df) or Z	*p*-value (effect size)	WODAA	PACE	t(df) or Z	*p*-value (effect size)	WODAA	PACE	t(df) or Z	*p*-value (effect size)
Total daily activity (steps)	5,992 ± 2,042	5,200 ± 2,298	*t* (47) = 1.3	0.217 (0.07)	**8,643 ± 3,265**	**5,564 ± 2,243**	***t* (33.6) = 3.7**	**.003** **(0.11)**	**1,912 [1,117–4,582]**	**451 [−309–1,106]**	***Z* = −3.7**	**0.000** **(0.27)**
Activity zone minutes (daily average)												
Sedentary (1MET, > 10 min)	1,072 [749–1,206]	1,066 [717–1,142]	*Z* = −0.1	2.8 (0.0)	977 [649–1,059]	1,050 [665–1,175]	*Z* = −1.3	0.62 (0.04)	−118 ± 229	−47 ± 161	*t* (47)=−1.3	0.207 (0.19)
Light (2–3 METs)	248 ± 69	228 ± 82	*t* (47) = 0.9	1.07 (0.02)	252 ± 62	224 ± 76	*t* (47) = 1.4	0.519 (0.03)	4 ± 57	−3 ± 44.0	*t* (47) = 0.5	0.617 (0.01)
Fairly active (4–6 METs)	14 [8–28]	22 [4–41]	*Z* = −0.5	1.95 (0.01)	29 [17–50]	22 [10–31]	*Z* = −1.6	0.36 (0.05)	**12 [3–31]**	**2 [−12–13]**	***Z* = −2.0**	**0.048** **(0.08)**
Very active (>6 METs)	8 [3–17]	7 [0–24]	*Z* = −0.40	2.1 (0.00)	**23 [13–48]**	**9 [4–21]**	***Z* = −3.3**	**0.02** **(0.22)**	**13 [6–34]**	**2 [−3–8]**	***Z* = −3.8**	**0.000** **(0.30)**
Heart Rate zone minutes (daily average)												
Fat burn (50–69% max HR)	159 [67–390]	340 [145–494]	*Z* = −2.1	0.078 (.09)	236 [98–460]	317 [177–507]	*Z* = −0.8	1.24 (0.01)	62 ± 82	2 ± 115	*t* (45) = 1.2	0.054 (0.04)
Cardio (70–84% max HR)	0 [1–10]	4 [1–8]	*Z* = −1.2	0.42 (0.03)	11 [4–25]	7 [3–11]	*Z* *=* −1.8	.132 (0.07)	**5 [2–22]**	**0 [−3–6]**	***Z* = −2.5**	**.011** **(0.14)**
Peak (85–100% max HR)	0 [0–0]	0 [0–0]	*Z* = −0.2	1.7 (0.00)	0 [0–2]	0 [0–0]	*Z* = −1.6	0.22 (0.06)	0 [0–2]	0 [0–0]	*Z* = −1.5	.145 (0.03)

Data are presented for each intervention group with significant differences between groups indicated in bold (*p* < 0.05). Values are presented as Median [25%–75% IQR] or as Average ± 1SD. Depending on the test, effect sizes are calculated using Cohen's d or *η*^2^.

### 3.2 Program retention & sustainability

Both PA regimens were generally well-tolerated by participants in both groups during the 4-Month intervention. Of the 65 individuals cleared for participation in the intervention following the initial assessments, 54 (24 WODAA, 30 PACE) completed the 4-month intervention and Final Assessment. The WODAA group had 6 participants that did not complete the intervention (1 due to illness, 3 due to loss of contact, 2 incompletions due to Covid-19 Pandemic protocols) while the PACE group had 4 participants that did not complete the intervention (2 due to exacerbation of previously unreported elbow and neck pain, 1 due to moving out of state, 1 due to loss of contact). Contact with one additional individual was lost prior to randomization.

Overall, 26 adverse events were reported, with 22 unlikely to be related to study participation. Of the remaining 4 adverse events that may be related to study participation, one event was minor, although directly attributable to the study (skin irritation from the activity monitor band, participant resumed after band replacement), and one was probably related (pain in elbow/biceps when lifting legs before transferring). The two possibly related events included a reaggravation of elbow pain from a participant with an initially undisclosed prior history and another with shoulder pain from increased arm cycling at high intensity in the absence of performing the recommended shoulder protection program.

### 3.3 Daily physical activity

#### 3.3.1 Steps

**Figure 2:**
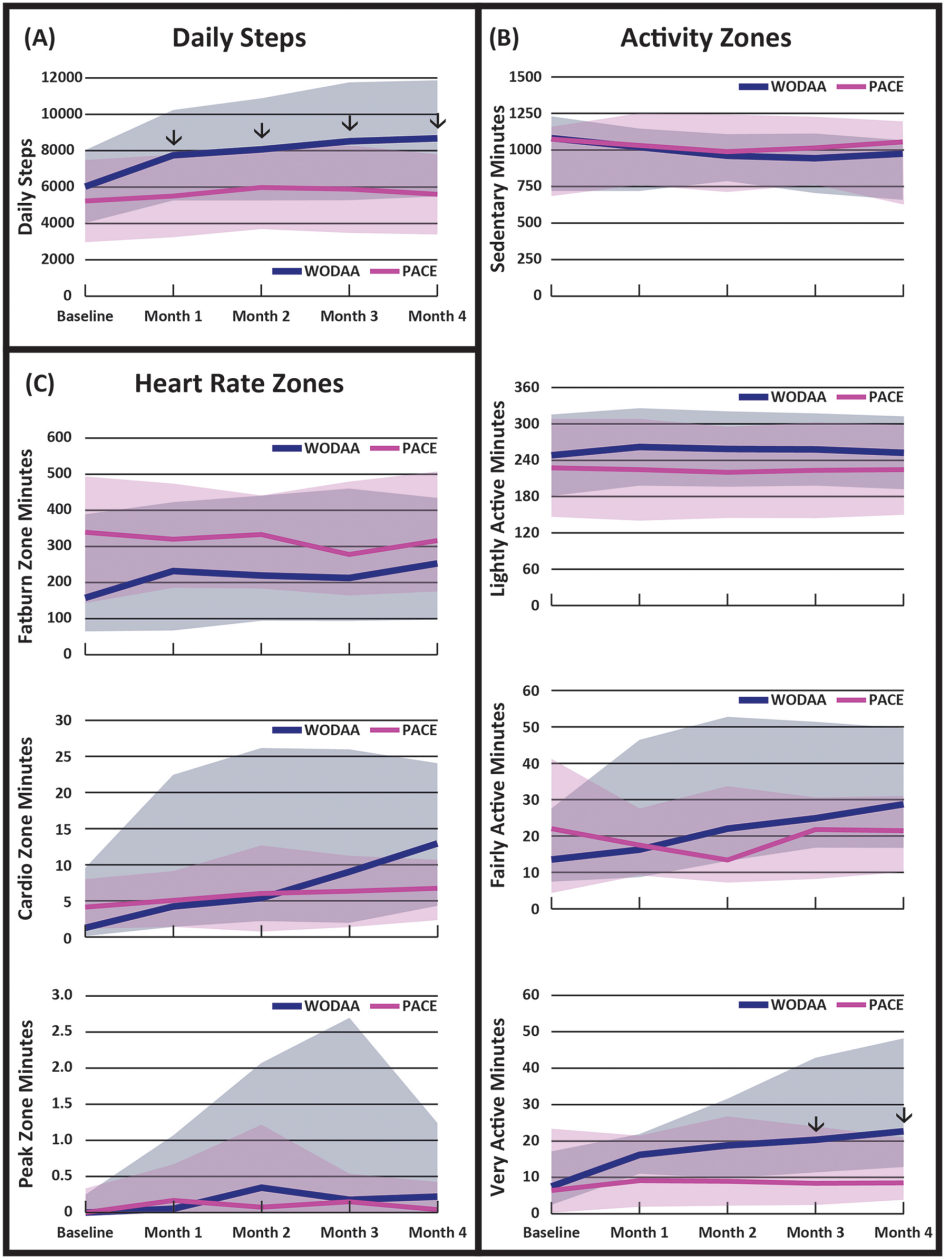
Daily averages of: **(A)** overall activity (steps), **(B)** minutes spent in different activity zones, and **(C)** minutes spent in different heart rate zones, obtained at five intervention assessment timepoints. Arrows (↓) denote time periods with significant differences (*p* < 0.05) between the WODAA and PACE groups. Please note the change in scales between variables. Daily step data are presented as means (lines) ± 1 standard deviation (shaded area). All other data are presented as median (lines) and 25–75 interquartile range (shaded area). Activity Zones were defined to be Sedentary (1MET, >10 min), Light (2–3 METs), Fairly Active (4–6 METs), and Very Active (>6 METs). Heart Rate Zones were defined as Fat Burn (50%–69% Max HR), Cardio (70%–84% Max HR), and Peak (85%–100% Max HR).

Significant (*p* < 0.05) increases in average daily Steps for each intervention month (1–4) were only observed in the WODAA group (Figure 2A, Table 3). In addition, the improvement in daily Steps between Baseline and Month 4 was significantly higher in the WODAA group compared to the PACE group {1,912 [1,117–4,582 Interquartile Range (IQR)], vs. 451 [−309–1,106 IQR], *p* = 0.000} (Table 2).

**Table 3:** Comparison of average total daily activity (steps ± 1SD) between the WODAA and PACE intervention groups.

Time	Average daily steps			
	WODAA	PACE	*t* (df)	*p*-value (effect size)
BL	5,992 ± 2,042	5,200 ± 2,298	*t* (47) = 1.3	0.217 (0.03)
Month 1	**7,706** **± 2,540**	**5,473** **± 2,305**	***t* (47) = 3.2**	**0.006** **(0.07)**
Month 2	**8,035** **± 2,870**	**5,942** **± 2,323**	***t* (47) = 2.8**	**0.021** **(0.06)**
Month 3	**8,483** **± 3,316**	**5,852** **± 2,450**	***t* (47) = 3.2**	**0.006** **(0.07)**
Month 4	**8,643** **± 3,265**	**5,564** **± 2,243**	***t* (34) = 3.7**	**0.002** **(0.11)**

BL, Baseline Period. Significant differences (*p* < 0.05) are presented in bold.

#### 3.3.2 Activity zone minutes

The WODAA group spent significantly more daily minutes compared to PACE in the Very Active Zone (approximately >6 METS) during both Month 3 [20 (12–43 IQR) vs. 9 (3–24 IQR); *Z* = −2.63, *p* = .027, effect size *η*2 = 0.14] and Month 4 [23 (13–48 IQR) vs. 9 (4–21 IQR); *Z* = −3.25, *p* = .022, *η*2 = 0.22] (Figure 2B, Table 2). Relative to the PACE group, the WODAA group had significantly greater improvements (compared to Baseline) in daily minutes spent in both the Fairly Active [12 (3–31 IQR) vs. 2 (−12–14 IQR); *p* = 0.048] and Very Active Zones [13 (6–34 IQR) vs. 2 (−3–8 IQR); *p* = 0.000; Table 2].

#### 3.3.3 Heart rate zones

The WODAA group had significantly greater improvement in daily minutes spent in the Cardio HR Zone from Baseline to Month 4 [5 (2–22 IQR) vs. 0 (−3–6 IQR) minutes; *p* = 0.011; Figure 2C, Table 2]. No other significant differences were observed within or between groups.

### 3.4 Cardiometabolic indicators

#### 3.4.1 6-Minute Push Test (6-MPT)

**Figure 3:**
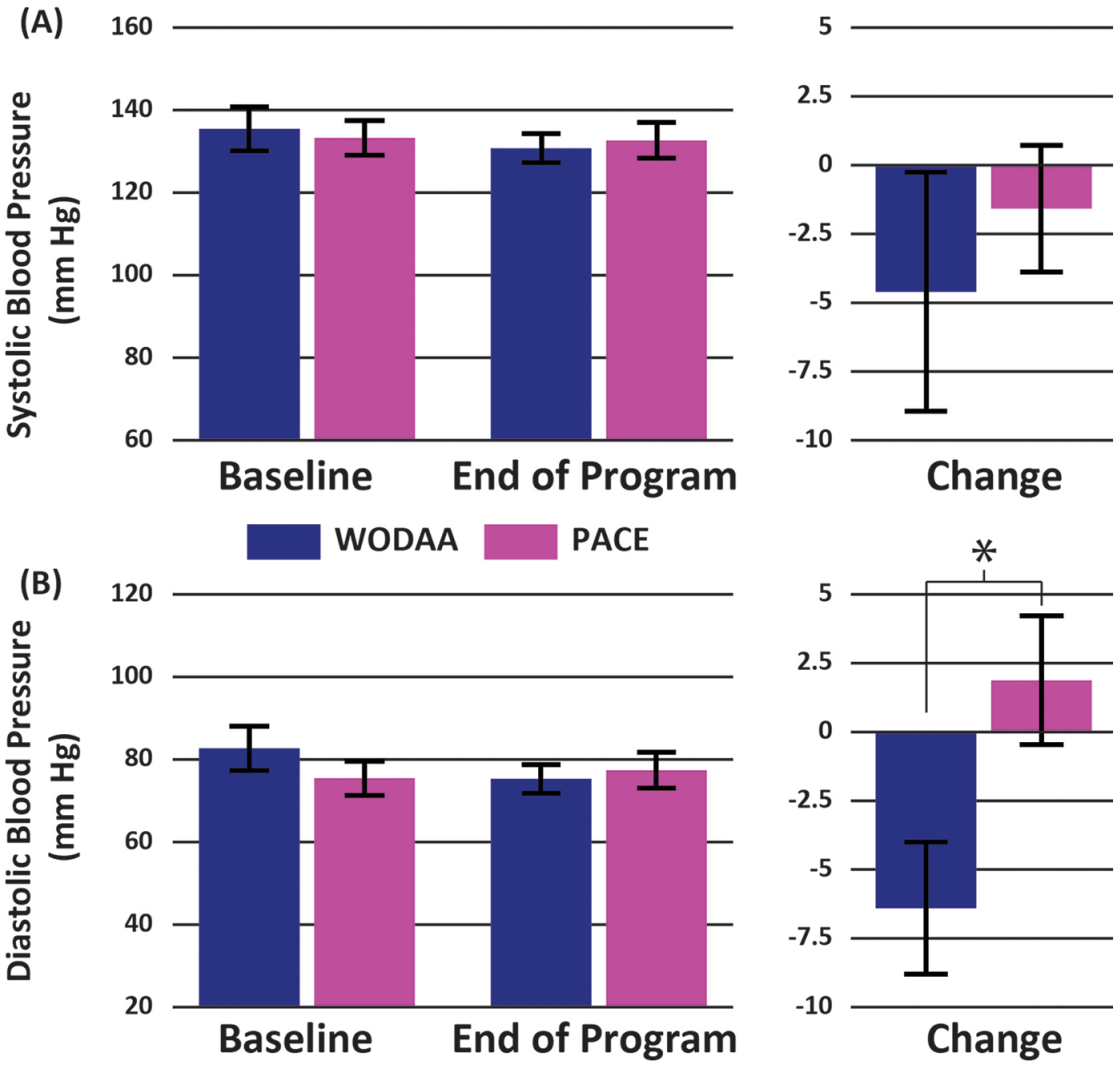
Group average Post 6-MPT **(A)** systolic and **(B)** diastolic blood pressure measurements for the WODAA and PACE groups at baseline and end of program (final assessment) timepoints (left column). Corresponding changes in values between the two timepoints are also presented (right column). All measurements were taken immediately following the completion of the 6-Minute Push Test. Error bars indicate Standard Errors. *Significant difference (*p* < 0.05) between the two intervention groups (WODAA, PACE).

The WODAA group demonstrated a significant decrease in Post 6-MPT Diastolic blood pressure following intervention (Baseline vs. Final Assessments) while PACE participants did not (−6 ± 11 vs. 2 ± 12; *t* = 1.8, *p* = 0.019; Figure 3). The resulting effect size, measured using Cohen's D, was 0.74, indicating a medium effect. No significant difference between intervention groups was observed in total distance pushed during the post-intervention 6-MPT assessment, with values for both groups similar to Baseline measurements (WODAA 727 ± 141 m post vs. 697 ± 126 m Baseline; PACE 718 ± 152 m post vs. 682 ± 176 m baseline; *p* ≥ 0.05).

#### 3.4.2 Fasting metabolic labs

No significant differences were noted between any metabolic laboratory values including HOMA index scores following either intervention (*p* > 0.05). Interestingly, relative to WODAA, there was a trend towards significantly lower Triglycerides at the Final Assessment in the PACE group [80 mg/dl (57–136 IQR) vs. 123 mg/dl (94–189 IQR), *Z* = −2.22, *p* = 0.052, *η*^2^ = 0.11] as well as a trend towards larger reductions in Triglyceride levels compared to baseline [−5 mg/dl (−29–9 IQR) vs. 7 mg/dl (7–46 IQR), *Z* = −1.85, *p* = 0.092, *η*^2^ = 0.06].

### 3.5 Wheelchair User's Shoulder Pain Index (WUSPI)

Shoulder Pain Baseline median WUSPI index scores were 0 [0–0 IQR] in both intervention groups and did not change significantly with either PA intervention (Table 1).

## 4 Discussion

This is one of the first studies directly comparing a WODAA program to a traditional PACE program for PA promotion in a group of MWC users with SCI. Aligning with our hypothesis, our findings suggest that a WODAA approach to increasing physical exercise is viable, accessible, and of greater or similar benefit than a PACE program for persons with SCI using MWCs. While both groups improved their overall activity compared to baseline measurements, our WODAA intervention helped participants significantly increase daily activity (Steps) and total time spent in higher intensity activities (Fairly, Very Active, and Cardio Zones) when compared to the PACE program. The WODAA program may also improve cardiovascular fitness as suggested by a small, but significant decrease in diastolic blood pressure measured immediately following 6-MPT at program end compared to baseline. Neither of the PA programs (WODAA and PACE), when combined with the STOMPS shoulder exercise program instruction, resulted in an onset of shoulder pain (i.e., no significant change in WUSPI scores). However, contrary to our hypothesis, there were no significant changes in fasting metabolic lab measures (lipids, insulin resistance, glucose), nor other fitness measures from the 6-MPT (meters pushed, Pre/Post-test systolic, Pre-test diastolic BP, Pre-test systolic BP), although there was a tendency towards lower triglycerides at program end in the PACE group. Despite the absence of significant improvements in most cardiovascular health measures, when considering WODAA's positive impact on increasing both overall activity (Steps) and higher intensity activity, there is sufficient evidence to suggest that a WODAA approach is a viable clinical alternative to traditional exercise programs.

Further, the WODAA approach to exercise may be especially relevant for those with limited access to resources required in traditional programs such as PACE. By using commercially available wrist-worn activity monitors to provide feedback on activity accumulated throughout the day, a WODAA program has the potential to address accessibility and affordability challenges to PA encountered by many individuals with SCI. Our findings, which document the WODAA intervention's effectiveness in a traditionally underrepresented community with limited-resources (>50% Hispanic, >75% low income; Table 1) represent a critical step towards identifying a potentially more accessible and equitable clinical prescription for increasing PA. In parallel, the advent of virtual and on-demand exercise programs – now widely available to the general population – can help overcome challenges in resource limited environments. However, few virtual/on-demand exercise programs tailored to MWC users and/or persons living with SCI currently exist and future work understanding how wearable sensors can be utilized to create and support these programs is needed.

A key component of both programs was the utilization of commercially based activity monitors to collect data and personalize PA programs for participants. While other research grade monitors may demonstrate higher accuracies, our approach that used a readily available and relatively low-cost activity monitor (Fitbit Blaze/Versa) was intentional: a primary consideration of the study was to evaluate the utility of commercially available activity monitors to promote PA in those with SCI, despite existing questions about data accuracy. While our overall outcomes were positive, suggesting that these devices can be used to promote PA, our findings should be interpreted within this context. The most obvious challenge associated with utilizing these activity monitors is that these devices do not account for the fundamental differences in physiological response (e.g., heart rate, metabolic energy expenditure) to exercise that exist between those living with an SCI and the general population (5, 40–42). In addition, these compromised responses will vary based on level and completeness of injury, with higher level and more complete injuries associated with greater differences in cardiovascular response and muscular capacity than those with lower and less complete injuries (5, 41, 42). Here we focused on assessing these devices with persons living with paraplegia (≤T2) to exclude the dramatically altered cardiovascular responses to exercise demonstrated by individuals with tetraplegia (5, 31). Of those studied, sixteen (16) individuals in this study had high paraplegia (T2–T6) and the remaining participants had low paraplegia (≤T7), with similar distributions between the two groups (Table 1).

Because individuals with paraplegia may also have varying levels of compromised cardiovascular response, we performed a *post hoc* analysis of our HR and blood pressure data obtained during the Baseline 6-Minute Push Test (6-MPT) to assess the validity of our maximum HR estimate, a key component to using commercial activity monitors. The analysis revealed no significant differences between individuals with high (T2–6) and low paraplegia (T7 and below) in maximum or average HR, total meters pushed, or peak and average HR when represented as a percentage of each participant's maximum HR obtained using an age-adjusted predictive equation (36). The only significant difference observed between groups was systolic blood pressure (BP) at assessment end. In this case, post-test systolic BP was reduced, on average, by 13.3 mmHg (9.6%) in those with T2-T6 paraplegia compared to subjects with lower-level injuries (high paraplegia; 124.6 ± 23.2 mmHg, low paraplegia; 137.9 ± 18.3 mmHg, *t* = 2.099, *p* = 0.041). Our *post hoc* findings largely fall in line with recent work studying relationships between injury level, maximal heart rate and age in eighty (80) individuals with thoracic SCI that demonstrated the traditional age-related decline in maximal HR is largely preserved, regardless of injury level (40).

With respect to the predictive equation for maximal HR used within our study, our estimates based on Tanaka, Monahan, and Seals (36) generated values that were 5–15 percent higher (depending on age) than those obtained using a predictive equation specific to individuals with low paraplegia that was published after our data collection (40). Despite this, we still believe that our findings related to differences in the amount of time spent in each HR Zone between groups are valid and contextually relevant. Because the maximum HR was likely overestimated for all individuals in our study, the Heart Rate Zones used in our analysis were also likely shifted upwards, resulting in a conservative approach that probably underestimated the amount of higher intensity PA performed by our participants, regardless of intervention group. This conservative approach strengthens our findings and overall conclusion that the WODAA program can be used to improve overall activity.

Similar to the HR Zones, Activity Zone data were determined using algorithms embedded within the commercially available activity monitor used in this study. In this case, each Activity Zone represents a predefined range of METS, calculated as the ratio of the current HR to resting HR. In this study, both the resting HR and activity-based HR were measured directly using the activity monitor. Because the underlying algorithms are designed for able-bodied individuals, the presentation of METS (and Activity Zones) obtained from these devices when studying those living with SCI should be done with caution. Fortunately, the validity and use of METS in this population has been studied previously, providing context for interpreting our findings (43). Using direct measurements of the metabolic cost of different activities in 170 individuals living with SCI (43), others have determined that the 1 MET equivalent for persons with SCI is lower than that of the general population (2.7 vs. 3.5 ml kg-1 min-1), with no significant difference in resting MET rates between different injury groups. Particularly relevant to the findings of this study, the authors proposed that arm crank exercises at medium to high intensities performed by those living with SCI correspond to a higher MET value relative to the general population (7.6 vs. 5.9 METS, respectively). Thus, like our HR Zone data, our presented Activity Zone data are likely to be conservative in nature and, as a result, significant increases in Activity Zones are likely underestimated.

For this study, we utilized a commercially available wrist-worn activity monitor (Fitbit Blaze/Versa) to record PA (Steps) and HR. At the time of data collection, the reliability and validity of these devices when measuring activity in wheelchair users (with or without SCI) had not been published. In preparation for this study, our pilot work demonstrated that the Fitbit Blaze/Versa was sufficiently accurate in recording the primary activities investigated in this study. More recently, the validity of such devices to document HR and energy expenditure have been widely investigated in able-bodied adults and, to a lesser extent, in MWC users (19, 26, 44–46). Importantly, the results of our pilot study are consistent with recent studies investigating the use of current commercially available activity monitors to measure HR and movement during MWC-based activities, with all findings supporting the notion that these devices can be used to support PA programs tailored for MWC users. For those with thoracic and lumbar SCI (T1–T5 and T6 and below), the Fitbit Charge 2 was found to have measurement errors in line with those we found in our pilot work (average errors of 6.2% and 4.1%, respectively for the two groups vs. 1.9%–4.9% in our pilot) when measuring HR during 11 different WC activities (26). These activities included arm-crank ergometry, where errors in HR measurements were found to be slightly higher, but still acceptable for the two groups (10% and 9.2%, respectively). A second study found that the Fitbit Versa reported lower HR during treadmill WC propulsion at 9 different intensities in WC users with physical disability (65% with SCI), although with a higher mean absolute percentage error (MAPE) of 17.4% (SD 12.4%) across conditions (45). In contrast, energy expenditure was greatly overestimated by the activity monitors in this study, averaging 71.2% across conditions (range 155.5 to 28.1%). However, MAPE consistently decreased with increasing intensity of propulsion, suggesting that the devices are better equipped to record high intensity activities such as PACE.

Though small (−6 mmHg), the significant decrease in diastolic BP after a bout of exercise at the end of the WODAA program supports the notion that our novel approach may improve cardiovascular health. For the general population, increased aerobic exercise has been associated with improved vasodilation and reduced vascular resistance, attenuating cardiovascular responses to stress post exercise and providing a cardioprotective benefit (47). However, this interpretation should be taken with caution as the number of studies that have systematically investigated how cardiovascular variables may change after a prescribed exercise program in those living with SCI are limited and have produced mixed results [e.g., (37, 48–50)]. While not studied here, this increased activity may hold further implications if this change persists over a long period of time, particularly if combined with positive nutritional changes. In contrast, there was no observed change in diastolic BP at program end for the PACE group (+2 mmHg). In addition, although not statistically significant, sedentary time in the WODAA group also had notable decreasing trends. When considered with the significant improvement of the post-6-MPT diastolic blood pressure, these two observations may indicate that the WODAA program can assist in establishing life-long habits of PA that, when sustained and coupled with the STOMPS shoulder exercise program, will provide long-term PA benefits to those in the program. However, while positive and significant PA changes were accomplished over 4 months, a longer intervention period may be needed to induce more measurable cardiovascular and metabolic health improvements in this population.

There are several established guidelines to help individuals obtain the physical benefits associated with activity and exercise. The CDC established weekly exercise guidelines for the general population of “150 min of moderate-intensity activity [3–5.9 METs] or 75 min of vigorous-intensity activity [>6 METs]” (35). Guidelines specific to those living with SCI have also been proposed, with recommendations typically covering cardiovascular health, muscle strengthening, and stretching (51–55). Suggested amounts for each type of exercise can vary greatly, with the largest range being in aerobic recommendations [from 20 min twice a week (52) to 30 min five times a week (54)]. Intriguingly, at Baseline, individuals in both groups consistently met the weekly exercise guidelines established by the CDC for the general population as well as most of the aerobic guidelines suggested for those living with SCI, despite our efforts to include only those that were not regularly exercising. The observed substantial levels of Baseline exercise may be partially due to participants knowing that they are being tracked and, therefore, increasing their sense of accountability to PA despite our efforts to minimize this effect by instructing them to avoid making any lifestyle changes during the baseline PA recording period. Even with this relatively high starting point in both intervention groups, however, the WODAA program still steadily and significantly increased PA (Steps) and time spent in higher Activity Zones (approximately ≥4–6 METS; Figure 2B), with observed increases in Fairly and Very Active Zone minutes, by the final month of intervention reaching 25 daily minutes (175 min/week). In contrast, PACE participants maintained similar activity levels throughout the intervention, suggesting a potential offsetting reduction of activity outside of PACE sessions. In addition, the larger decrease in daily sedentary time of 118 min in WODAA as compared to 47 min in PACE, while not statistically significant, may still hold import in addressing morbidity and mortality disparities amongst those with SCI who are also low-active (<2 h of activity per day), particularly if sustained over a longer duration. In fact, others have found that replacing 30 min of daily sedentary time with an equal amount of at least light activity time, as WODAA participants accomplished, was associated with a 20% reduction in mortality risk after 5 years, while a 39% mortality risk reduction was noted when replacing an hour daily of sedentary time [or non-activity] with an hour of exercise light activity among low-active individuals (56–58).

### 4.1 Limitations and future recommendations

The results of the current investigation must be considered alongside the limitations of the study. While our pilot work and current literature suggests that commercially available activity monitors may be adequate for measuring activities performed by MWC users, the devices used in this study were not specifically designed for this purpose. However, a recent meta-analysis of Fitbit devices determined that the specific Fitbit model is not a significant factor when evaluating validity evidence of these devices (44), suggesting that the specific devices used in our study would have performed similarly to those evaluated in more recent validity tests. Regardless, documenting arm movements (as Steps), HR, and energy expenditure with these devices will introduce varying amounts of error dependent on the nature and intensity of the task. These errors would have presented across all participants within both interventions, minimizing their overall influence on study outcomes. In addition, the large differences in recorded activity levels between WODAA and PACE (e.g., average of 2,287 daily Steps at Month 4) suggests that any variance in recording accuracy would account for only a small portion of the observed change between groups. A second consideration is that the age-adjusted estimation of maximum HR used in this study was based on healthy adults without SCI. The equation used in this study (36) resulted in overestimation of max HR for our participants with paraplegia. Future work should utilize more accurate equations that were unavailable at the time of our data collection such as those proposed by Hamner and colleagues (40). Last, the WODAA group received constant feedback from their activity monitor while the PACE group received only HR feedback during PACE sessions. As a result, our study design did not allow us to determine how the two different levels of biofeedback (constant vs. minimal) may have influenced participant performance, including its influence on the observed decrease in PA outside of arm cycling sessions in the PACE group. Despite these limitations, our work represents what we believe would be a typical implementation of activity monitors to support both interventions and accurately represents the associated outcomes.

## 5 Conclusion

This study evaluated the viability of a whole of day activity accumulation (WODAA) approach PA intervention that uses a commercially available wrist-worn activity monitor for biofeedback. Depending on the measure used, the current study documents this approach to be more effective or provide similar PA improvements when compared to a traditional planned arm crank ergometry (PACE) program. Equally important, our results demonstrate our underlying assumption that using commercial activity monitors can be effective for promoting PA in MWC users with paraplegia is valid. When using a commercial activity monitor, we found that WODAA group participants demonstrated steady improvements throughout the program, including significant increases in daily arm (pushing) activity, time spent in moderate to vigorous intensity activities (≥4–6 METS), and time spent in the Cardio HR Zone (70%–84% of participants' predicted maximum HR) by program end (Month 4). As a result, participants in this group met or exceeded established exercise guidelines during the final intervention month. In contrast, those engaged in the traditional PACE program did not significantly increase overall PA, with ergometry sessions inducing a likely offsetting reduction in non-exercise activity. However, because these monitors are not tailored to account for the potential of a compromised cardiovascular response in those living with SCI, overall activity may be underestimated in both groups. Neither group experienced increases in shoulder pain, suggesting that, when appropriately implemented with shoulder pain prevention exercises (e.g., STOMPS), both PA programs can be implemented without increasing the likelihood of participants experiencing deleterious effects on shoulder health and function.

Future investigations to determine the impact of adding real-time PA feedback to a PACE intervention, pairing nutritional interventions to evaluate the effect that longer PA interventions may have on cardiometabolic health, and to evaluate alternative approaches for measuring activity levels in those with more compromised cardiovascular responses to exercise are critical moving forward. Results from this study may help healthcare providers and persons with SCI using MWCs for locomotion make informed decisions in choosing effective interventions to increase PA, particularly for those with limited resources, potentially impeding access to a traditional PACE program.

## Data Availability

The raw data supporting the conclusions of this article will be made available by the authors upon request, without undue reservation.
